# A guide to understanding and measuring photosynthetic induction: considerations and recommendations

**DOI:** 10.1111/nph.70218

**Published:** 2025-06-01

**Authors:** Liana G. Acevedo‐Siaca, Lorna McAusland

**Affiliations:** ^1^ Horticulture and Product Physiology Wageningen University 6708 PB Wageningen the Netherlands; ^2^ Division of Plant and Crop Sciences, School of Biosciences University of Nottingham Leicestershire LE12 5RD UK

**Keywords:** acclimation, light, photosynthesis, photosynthetic induction, Rubisco, stomatal conductance

## Abstract

Photosynthetic induction is the leaf‐level process by which a plant assimilates CO_2_ from the atmosphere once exposed to a change in light intensity after a period of darkness or shade. In the field, photosynthetic induction can take place hundreds of times in a single day in response to rapid fluctuations in the light environment due to cloud cover, wind, solar angle, and neighbourly shading. In general, the speed of photosynthetic induction is broadly regulated by two main components: the diffusional limitations of CO_2_ reaching the sites of carboxylation; and the biochemical limitations associated with the assimilation of CO_2_. Quantifying these limitations and exploring genetic diversity can lead to the optimization of photosynthetic efficiency, and consequently, increased plant productivity. Growing numbers of studies have shifted away from characterizing photosynthesis in steady‐state light environments in preference to understanding photosynthetic induction under more realistic, dynamic light environments. In this guide, we aimed to promote consistency between studies and facilitate comparison of results with and cross species by: discussing best practice when designing an experiment focussed on measuring photosynthetic induction; providing resources for analysing photosynthetic induction data; and identifying gaps in our collective knowledge relating to photosynthetic induction.

**Table:** 

	Contents	
	Summary	450
I.	Introduction: what is photosynthetic induction and when does it happen?	450
II.	Measurement considerations: designing a photosynthetic induction experiment	453
III.	Quantifying photosynthetic induction; calculating limitations and rate of response	458
IV.	Future steps	463
V.	Conclusions	464
	Acknowledgements	465
	References	465

## I. Introduction: what is photosynthetic induction and when does it happen?

Plants grow and live in dynamic environmental conditions, in which they must respond, adapt, and acclimate to changes in temperature, water availability, and light. Light conditions within plant canopies are rarely constant. Instead, leaves inside the canopy must cope with periods of shade and full sun, which may last between fractions of a second and several minutes. Depending on their duration, these changes in light intensity – or photosynthetic photon flux density (PPFD) – can be categorized as different types of lightflecks and can occur due to changes in cloud cover, wind, sun angle throughout the day, and genotypic canopy architecture (Pearcy, 1990; Way & Pearcy, 2012; Durand & Robson, 2023; Sellaro *et al*., 2024). Even on clear, still days, a leaf may experience hundreds of lightflecks lasting anywhere between < 10 and > 120 s over the course of the entire day (Way & Pearcy, 2012). Consequently, this fluctuating light environment plays a vital role in plant productivity and is estimated to contribute between 10 and 80% of the PPFD available for photosynthesis for understory leaves (Pfitsch & Pearcy, 1989; Chazdon & Pearcy, 1991; Leakey *et al*., 2005).

Our understanding that plants grow in dynamic light environments is not new (Pearcy & Way, 2012). Indeed, lightflecks were identified as a significant source of light throughout plant canopies, which vary throughout the day and through ecosystems, as early as the 1920s (Allee, 1926). Seeking to understand how lightflecks influence plant physiology, various studies throughout the 1970–1990s sought to characterize light fluctuations and their utilization in photosynthesis (Norman *et al*., 1971; Pfitsch & Pearcy, 1989; Pearcy, 1990; Chazdon & Pearcy, 1991). Work during this time period further elucidated the process of photosynthetic induction, the activation of Rubisco by Rubisco activase (Rca), and the impact of diffusional limitations on photosynthetic response to changing light (Leegood & Walker, 1980; Salvucci *et al*., 1985; Kirschbaum & Pearcy, 1988; Pearcy *et al*., 1996). This work was facilitated through the development of newer, faster infra‐red gas analyzers (IRGA) that could be used to characterize more dynamic photosynthetic processes (Pearcy & Way, 2012). However, despite plants growing in a constantly changing light environment and great advancements in measuring and replicating lightflecks, the majority of our understanding of photosynthetic processes is still within the context of steady‐state conditions.

Initial work on photosynthetic induction was heavily focussed on understory species, especially lower growing trees, shrubs, and herbaceous plants (Table 1). However, with time, the focus of photosynthetic induction studies shifted increasingly towards annual crop plants mostly grown in monoculture cropping systems (Table 1). Through this work, a growing body of literature suggests that measurements of photosynthesis in steady‐state conditions may be overlooking fundamental processes that can only be captured within the context of fluctuating light conditions (McAusland *et al*., 2016; Long *et al*., 2022). This includes natural variation for parameters known to limit photosynthetic performance, such as the induction state (IS) or speed of stomatal opening, that may not be captured in steady‐state measurements (Driever *et al*., 2014; Acevedo‐Siaca *et al*.,  2021a,b). Additionally, most photosynthetic research has focussed on steady‐state rates of *A* under light saturating conditions (*A*_sat_), which can be indicative of photosynthetic capacity, but is not representative of most ‘real‐world’, growing conditions of leaves within canopies. These kinds of measurements can also lead to an overestimation of diurnal photosynthesis of up to 3% on cloudy days to 30% on sunny days with many lightflecks (Pfitsch & Pearcy, 1989). Furthermore, depending on crop and environmental conditions, the magnitude of *A*_sat_ is not always positively correlated with yield, which could be related to the way in which photosynthesis has conventionally been measured (Sinclair *et al*., 2019; Weiner, 2019). However, inter‐ and intraspecific variation in the speed of crop photosynthetic induction suggests that the optimization of photosynthesis for fluctuating light conditions has not yet occurred (Wang *et al*., 2020). As a result, interest in measuring photosynthesis in fluctuating light conditions has increased in necessity and popularity, to begin to address questions left unanswered by steady‐state measurements.

**Table 1:** An overview of mixed and species‐specific publications on photosynthetic induction to light across different species for the three major photosynthetic types.

Photosynthetic type	Species	References
C_3_	*Varied species*	
	**–**	Ögren & Sundin (1996); McAusland *et al*. (2016)
	*Crops*	
	Wheat – *Triticum aestivum*	Kobza & Edwards (1987); Taylor & Long (2017), Townsend *et al*. (2018), Salter *et al*. (2019)
	Rice – *Oryza sativa*	Taniyoshi *et al*. (2020); Acevedo‐Siaca *et al*. (2020); Yamori *et al*. (2020), Acevedo‐Siaca *et al*. (2021a, 2021b); Sakoda *et al*. (2021)
	Soya bean – *Glycine max*	Soleh *et al*. (2016, 2017); Wang *et al*. (2020); Sakoda *et al*. (2021)
	Cassava – *Manihot esculenta*	De Souza *et al*. (2020)
	Tobacco – *Nicotiana tabacum*	Gómez *et al*. (2018)
	Barley – *Hordeum vulgare*	McAlister (1937)
	Cotton – *Gossypium hirsutum*	Han *et al*. (2022), Parkash *et al*. (2024)
	Cucumber – *Cucumis sativus* L.	Sui *et al*. (2011)
	Tomato – *Solanum lycopersicum*	Zhang *et al*. (2018); Sun *et al*. (2022); Sun *et al*. (2023)
	Pepper – *Capsicum annuum*	Wen *et al*. (2023)
	Spinach – *S. oleracea*	Prinsely & Leegood (1986); Fan *et al*. (2007)
	Brassica crops – *Brassica rapa*, *Brassica oleracea*, *Brassica napus*	Taylor *et al*. (2020)
	Collection of horticultural crops	Zhang *et al*. (2022)
	*Non‐crops, herbaceous*	
	Collection of species	Deans *et al*. (2019)
	Arabidopsis – *Arabidopsis thaliana*	Alter *et al*. (2012), Carmo‐Silva & Salvucci (2013); Kaiser *et al*. (2015, 2017); Sakoda *et al*. (2020)
	*Tree species*	
	Collection of species	Poorter & Oberbauer (1993); Tinoco‐Ojanguren & Pearcy (1993); Valladares *et al*. (1997); Hull (2002); Naumburg & Ellsworth (2002), Timm *et al*. (2002), Leakey *et al*. (2005); Urban *et al*. (2007); Way & Pearcy (2012); Kang *et al*. (2020)
	*Shorea leprosula*	Leakey *et al*. (2003)
	*Populus trichocarpa*	Han *et al*. (2022)
C_4_	*Crops*	
	Maize – *Zea mays*	Long *et al*. (1983); Chen *et al*. (2012); Qiao *et al*. (2021), Wang *et al*. (2021)
	Sorghum – *Sorghum bicolor*	Wang *et al*. (2021); Pignon *et al*. (2021a, 2021b)
	Sugar Cane – *Sachhar officinarum*	Wang *et al*. (2021)
	Miscanthus – *Miscanthus giganteus*	Ubierna *et al*. (2013); Sun *et al*. (2014)
	*Non‐crops, herbaceous*	
	*Flaveria bidentis*	Ubierna *et al*. (2013)
	*Microstegium viminium*	Horton and Neufeld (1998)
	Common Cordgrass – *Spartina angelica*	Long (1976, 1983)
CAM	*Bryophyllum pinnatum*	Yang *et al*. (2019)
	*Mesembryanthemum crystallinum*	He *et al*. (2020)

**Fig. 1:**
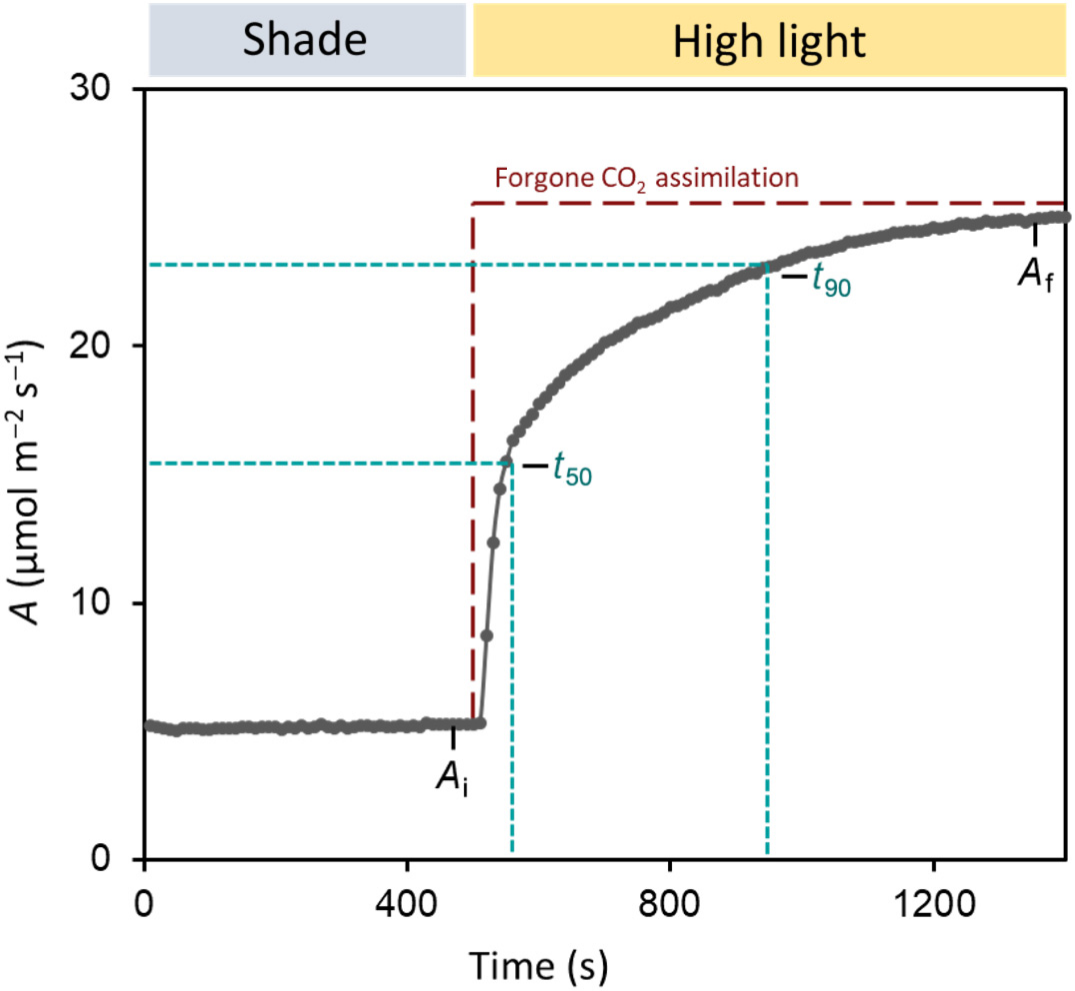
Photosynthetic induction describes the increase in photosynthetic CO_2_ assimilation (*A*) in response to a change from low irradiance or shade to high irradiance. *A*_i_ is the initial CO_2_ assimilation rate during low irradiance, *A*_f_ is the final CO_2_ assimilation rate during high irradiance, t_50_ is the time to reach 50% of A_f_ during induction, t_90_ is the time to reach 90% of *A*_f_ during induction, and forgone CO_2_ assimilation (dashed section in red) represents the lost potential CO_2_ assimilation during photosynthetic induction relative to *A*_f_ due to a lag in photosynthetic efficiency. Data shown are from Acevedo‐Siaca *et al*. (2020) for rice (*Oryza sativa*) (accession no. IR64‐21).

Photosynthetic induction is the process by which plants or leaves begin to assimilate CO_2_ upon being exposed to an increase in light intensity following a period of shade or darkness. Photosynthetic induction is characterized by a lag in photosynthetic efficiency during the response from low light to high light. Initially, CO_2_ assimilation (*A*) responds almost instantaneously to the change in PPFD; however, it can take several minutes to achieve final, steady‐state photosynthetic rates (Fig. 1). The lag in efficiency during induction is caused by several concurrent limitations that the leaf must overcome in order to assimilate CO_2_, including the following: diffusional limitations related to slow stomatal opening and mesophyll conductance; the activation of Rubisco by Rca; the photoactivation of enzymes involved in the regeneration of ribulose 1,‐5 bisphosphate (RuBP); and the build‐up of carbon metabolism intermediates involved during the Calvin–Benson–Bassham (CBB) cycle (McAusland *et al*., 2016; Busch *et al*., 2020; Sakoda *et al*., 2021; Liu *et al*., 2022). The rate or speed of photosynthetic induction – as well as its different limitations – is genotype‐ and species‐specific (Auchincloss *et al*., 2014; Wachendorf & Küppers, 2017; Yamori *et al*., 2020). In addition to the aforementioned limitations, the increase in CO_2_ assimilation during photosynthetic induction is coupled with the induction of nonphotochemical quenching (NPQ), which dissipates any excess absorbed energy that can otherwise result in photoinhibition and/or the generation of reactive oxygen species (Murchie & Ruban, 2020). Nonphotochemical quenching may also present a unique limitation to photosynthetic induction during nonsaturating light fluctuations in leaves that have previously been exposed to a period of high irradiance. In this case, there may be excessive NPQ relative to the amount of photochemistry taking place.

In crop plants, reducing or overcoming the limitations that impede quick CO_2_ assimilation during photosynthetic induction presents a significant opportunity to improve overall productivity and yields (Fig. 1) (Taylor & Long, 2017; Long *et al*., 2022). For example, it is estimated that inefficient photosynthetic induction in wheat may cause penalties to crop productivity of 21% compounded over the course of a growing season (Taylor & Long, 2017). In important staple crops such as sorghum, soya bean, rice, and cassava, previous studies have identified significant inter‐ and intraspecies natural variation for traits related to photosynthetic induction, both in the speed of induction and in the amount of CO_2_ assimilated during induction (McAusland *et al*., 2016; Soleh *et al*., 2016; Acevedo‐Siaca *et al*., 2020, 2021a,b; De Souza *et al*., 2020; Yamori *et al*., 2020; Battle *et al*., 2024). In general, variation in the rate of photosynthetic induction is lower between members of the same species and greater between species (McAusland *et al*., 2016). Additionally, differences were identified in the coordination between stomatal opening and CO_2_ uptake (McAusland *et al*., 2016), which could help to reduce water loss through stomata in fluctuating light conditions. These studies suggest that combinations of these traits may have sufficient natural variation to be amenable to modification, selection, and improvement. However, to achieve this goal, studies need to be expanded to more crops and genotypes. Until now, most studies evaluating photosynthetic induction in crop plants focus on C_3_ plants, but less work has focussed on crops or species with alternative photosynthetic pathways – including C_2_, C_4_, and crassulacean acid metabolism – offering a significant opportunity to expand our knowledge about the process in distinct carbon fixation pathways (Lundgren, 2020; Wang *et al*., 2021; Tanigawa *et al*., 2024).

For noncrop plants in ecological niches, such as trees and herbaceous plants, the ability to maximize carbon uptake in these short interludes of high light determines the growth and survival of plants within their environment (Way & Pearcy, 2012; Smith & Berry, 2013). The duration and properties of lightflecks are partially dependent upon the canopy depth and species composition of the ecosystem, both of which also affect the spectral composition within their environment (Durand *et al*., 2021; Durand & Robson, 2023). However, with increasing duration and intensity, the utilization of lightflecks may become secondary to other environmental stressors such as high leaf temperature, water availability, and increased photoinhibition (Leakey *et al*., 2005; Murchie & Niyogi, 2010; Smith & Berry, 2013).

Rather than summarizing recent and historical advances in the field of ‘dynamic’ photosynthesis, we offer a practical guide with factors to take into consideration when designing and executing experiments that aim to study photosynthetic induction utilizing techniques in infra‐red gas analysis. Historical and contemporary advancements in knowledge related to photosynthetic induction have been reviewed in great detail in recent years (Kaiser *et al*., 2019; Long *et al*., 2022), corresponding with renewed interest in the field of study. Many studies focussing on photosynthetic induction have been published in recent years, often using different experimental design criteria and adaptation of methods. This makes comparison between recent studies very difficult – in some cases impossible – and limits the ability to detect trends across species or ecological niches for traits related to photosynthetic induction.

Here, we present a practical guide to measuring photosynthetic induction in higher plants that: discusses the underlying limitations to photosynthetic induction; examines considerations when designing an experiment focussed on photosynthetic induction; and presents methods to analyze and interpret photosynthetic induction data.

## II. Measurement considerations: designing a photosynthetic induction experiment

**Fig. 2:**
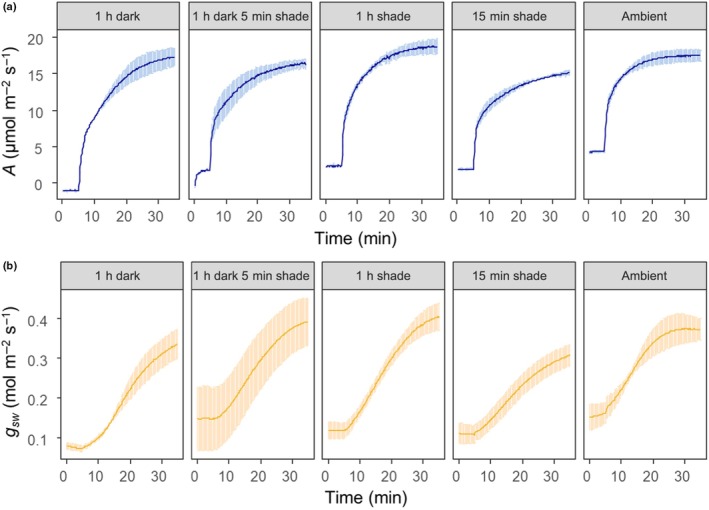
Photosynthetic induction responses of (a) CO_2_ assimilation (*A*) and (b) stomatal conductance of water (*g*_sw_) with different darkness or low irradiance adaptation periods before photosynthetic induction with high irradiance. The high irradiance level for all of the induction curves was 1800 μmol m^−2^ s^−1^. The five treatments were as follows: 1 h of darkness (0 μmol m^−2^ s^−1^) with photosynthetic induction directly from darkness, 1 h of darkness (0 μmol m^−2^ s^−1^) followed by 5 min of deep shade (50 μmol m^−2^ s^−1^) before photosynthetic induction, 1 h of deep shade (50 μmol m^−2^ s^−1^) before photosynthetic induction, 15 min of deep shade (50 μmol m^−2^ s^−1^) before photosynthetic induction, and photosynthetic induction directly from ambient light intensity (100 μmol m^−2^ s^−1^). The ambient light intensity is representative of the light intensity in which the plants were grown in the climate chamber. Data presented are from tobacco (*Nicotiana tabacum*) cv Petit Havana (*n* = 3). Error bars represent ± SE.

Environmental factors including light environment, temperature, water availability, and [CO_2_] all contribute to the rate of photosynthetic induction in response to step increases in light. In this section, we describe these factors within the context of measuring photosynthetic induction and some considerations regarding experimental design (Fig. 2).

### 1. The light environment

**Fig. 3:**
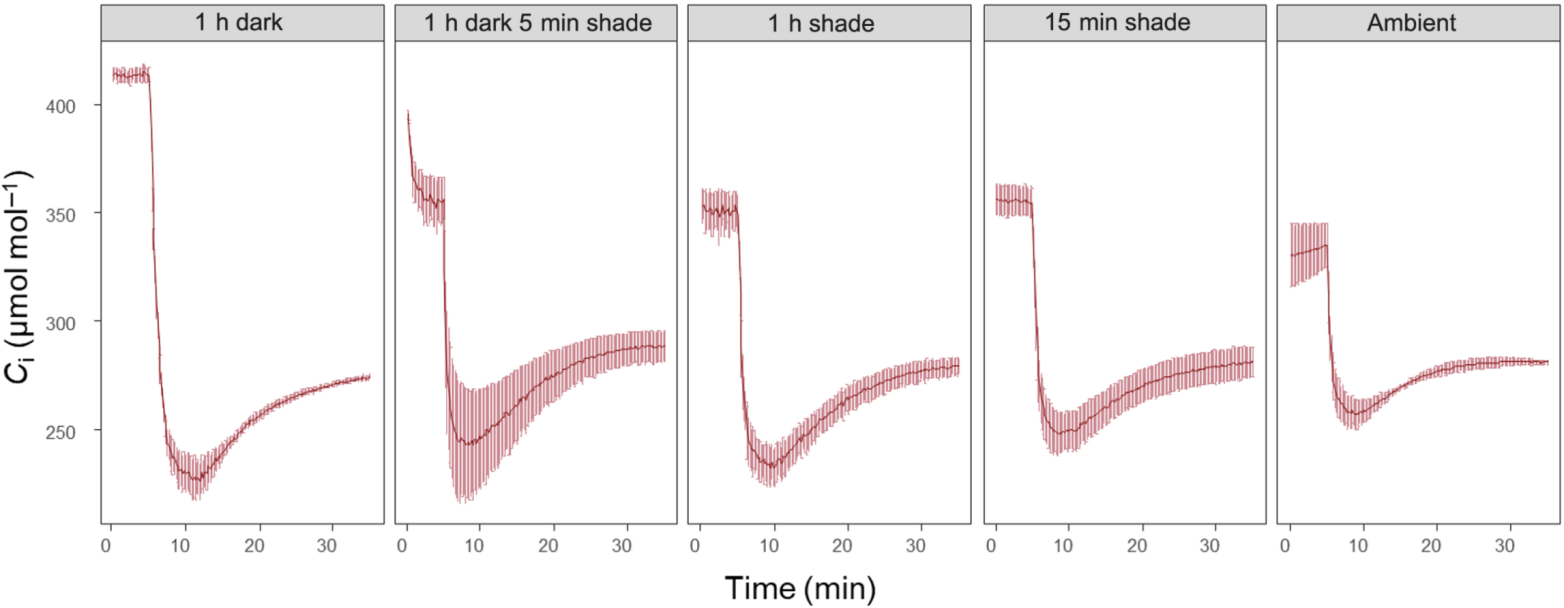
Photosynthetic induction responses of intercellular CO_2_ concentration (*C*_i_) with different darkness or low irradiance (photosynthetic photon flux density – PPFD) adaptation periods before photosynthetic induction with high irradiance. The high irradiance level for all of the induction curves was 1800 μmol m^−2^ s^−1^ PPFD. The five treatments were as follows: 1 h of darkness (0 μmol m^−2^ s^−1^) with photosynthetic induction directly from darkness, 1 h of darkness (0 μmol m^−2^ s^−1^) followed by 5 min of shade (50 μmol m^−2^ s^−1^ PPFD) before photosynthetic induction, 1 h of shade (50 μmol m^−2^ s^−1^ PPFD) before photosynthetic induction, 15 min of shade (50 μmol m^−2^ s^−1^ PPFD) before photosynthetic induction, and photosynthetic induction directly from ambient light intensity (100 μmol m^−2^ s^−1^ PPFD). The ambient light intensity is representative of the light intensity in which the plants were grown in the climate chamber. Data presented are from tobacco (*Nicotiana tabacum*) cv Petit Havana (*n* = 3). Error bars represent ± SE.

For most experiments documented in the literature, measuring photosynthetic induction typically consists of two steps: (i) exposing the leaf to low‐light levels, followed by (ii) exposure to high light, most often through a stepwise increase in light intensity. In many experiments, a dark adaptation or prolonged shade exposure of leaves is applied before measuring photosynthetic induction to ensure measurements are made from a known consistent or steady‐state set of measurements. This period of time enables many processes within the leaf to briefly acclimatize to these conditions, including stomatal conductance, and also facilitates comparable assessment of the kinetics of the induction. This period of stability may be also relevant, dependent upon the study, to ensure that residual NPQ is not playing a limiting role during photosynthetic induction. The rate of photosynthetic induction and final steady‐state photosynthesis are greatly influenced by the initial light environment the plant or leaf is exposed to (Figs 2, 3), underscoring the importance of taking initial light environment into consideration during experimental design.

Measuring photosynthetic induction directly from dark‐adapted leaves adds greater biochemical and diffusional limitations and is only representative of a narrow set of environmental conditions. It is generally recommended that leaves should be acclimated to a low‐light or ambient light intensity before being exposed to high‐light conditions. An exception for directly measuring from dark‐adapted leaves could be in experiments that aim to look at photosynthetic responses within the context of controlled environments (e.g. vertical farming), in which plants are often grown under square lighting and undergo a stepwise change in irradiance from darkness. In addition, studies investigating dawn or dusk where changes in light intensity are gradual may also need to include a period of darkness to fully investigate the limitations at these specific diurnal periods (Servaites *et al*., 1989; Annunziata *et al*., 2018).

Exposing the leaf to a period of low‐intensity light (typically 50–150 μmol m^−2^ s^−1^ PPFD) is sufficient to induce consistent pools of RuBP and Rubisco activation as well as stimulating initial stomatal opening to compare between genotypes and treatments (Matthews *et al*., 2020). As the intensity and duration of low‐light exposure can also influence the rate of induction (Sassenrath‐Cole & Pearcy, 1994), generally the duration of low‐light exposure is given as the time taken for stomatal conductance to reach steady‐state before the measurement of photosynthetic induction, which can take between 5 and 40 min, depending on leaf, genotype, and species (McAusland *et al*., 2016).

For the high‐light step, the intensity should be saturating (or near‐saturating) and not excessive enough to cause photoinhibition (Pearcy & Sims, 1994). For most crop species, this can be in the range of 1000–1800 μmol m^−2^ s^−1^ PPFD. However, saturating light can be determined for individual experiments through the measurement of light–response curves (Evans *et al*., 1993; see Busch *et al*., 2024 on guide to measuring light–response curves) and should be determined for the individual species and for its acclimated growing conditions before measuring photosynthetic induction.

When determining the magnitude and duration of the step increase from low to high light, it is recommended that the user consider which limitations are being assessed and when. For example, is the study's focus on understanding limitations to induction in the early or later stages of photosynthetic induction? In natural environments, the duration of lightflecks varies between milliseconds and a few minutes (Smith & Berry, 2013); however, this may not be long enough to determine the separate limitations of photosynthetic induction given the restrictions of measuring changes in CO_2_ assimilation and stomatal conductance on a millisecond to seconds timescale. Assuming steady‐state shade or dark adaptation, the activation of CBB cycle enzymes occurs within the first 1–2 min of induction after exposure to high light, while limitation by stomata can be less important (Pearcy, 1990; Acevedo‐Siaca *et al*., 2020). Meanwhile, full activation of Rubisco by Rca can take longer and has been documented to take between 2 and 4 min in wheat and up to 7 min for some horticultural crops (Pearcy, 1990; Salter *et al*., 2019; Zhang *et al*., 2022). Leaves are often left at high light to achieve a steady‐state *A*, which can take anywhere between 15 and 60 min depending on the species measured and is useful in later calculations of biochemical or diffusion limitations or for determining photosynthetic capacity. In this later period, the rate of stomatal opening can become a more important limitation to CO_2_ assimilation (McAusland *et al*., 2016; Acevedo‐Siaca *et al*., 2020).

Finally, acclimation to the light environment should be considered for every experiment. For example, plants that are grown in the glasshouse or field will be exposed to a higher degree of fluctuation throughout their lifetime than plants grown in a climate‐controlled growth room. Vialet‐Chabrand *et al*. (2017) have shown that Arabidopsis grown under square wave light intensities with no fluctuations – similar to those commonly found in growth rooms – demonstrated greater photosynthetic capacity than plants grown under realistic dynamic fluctuating conditions. This work underscores that the same species grown under nonfluctuating conditions were not representative of plants grown under conditions mimicking changes in diurnal light intensity (Vialet‐Chabrand *et al*., 2017; Deguchi & Koyama, 2020). Similarly, planting density in the field can significantly alter the intensity and number of lightflecks experienced by a crop throughout its lifespan (Burgess *et al*., 2017; Townsend *et al*., 2018; Zheng *et al*., 2023).

### 2. Temperature

In the field, increases in light are commonly accompanied by increases in temperature. Measuring and interpreting leaf‐level responses to this codependent change in environment is complex (Kang *et al*., 2020).

Leaf temperature is not only determined by air temperature but is also dictated by active processes (e.g. stomatal conductance) and leaf properties (e.g. hairs, thickness, colouration, and surface deposits). In the field, leaves at the top of a canopy may experience greater temperatures than leaves within the canopy due to exposure to higher longwave (thermal) radiation from sunlight. In the laboratory or in a controlled environment growth room, leaves may not experience the same magnitude of heat due to the lack of this radiation from modern grow lighting.

Leaf temperature is critical to photosynthetic processes, most notably enzyme functionality (Sage *et al*., 2008; Moore *et al*., 2021). In general, the rate of photosynthetic induction increases with temperature until *c*. 35–40°C (Yamori *et al*., 2014; Kaiser *et al*., 2015). At extremely high temperatures (≥ 40°C), the rate of induction can be negatively impacted due to the sensitivity of Rubisco and Rca to heat (Crafts‐Brandner & Salvucci, 2000; Jensen, 2000; Cavanagh *et al*., 2023) and the rate of regeneration of RuBP. As temperatures climb, the ratio of carboxylation to oxygenation decreases, leading to increased photorespiration and a decline in CO_2_ assimilated. While the primary role of Rca is to remove inhibiting sugars from the active site of Rubisco (Jensen, 2000), Rca undergoes conformational changes and can bind with thylakoid‐bound polysomes at high temperatures (Rokka *et al*., 2001). This is likely to protect the protein synthesizing machinery that is associated with the thylakoid from heat stress (Rokka *et al*., 2001). However, this results in a decrease in the rate of induction, as the rate of Rubisco deactivation exceeds the rate of activation by Rca (Crafts‐Brandner & Salvucci, 2000).

Such work has led to a greater understanding of the optimal quantity of Rca for Rubisco activation, whether the abundance of Rca isoforms affects temperature sensitivity, and how the thermostability of Rca can be improved (Salvucci & Crafts‐Brander, 2004; Yamori & von Caemmerer, 2009; Degen *et al*., 2020, 2021). For example, a decrease in Rca of up to 55% was found to not significantly alter the temperature sensitivity of Rubisco activation or the catalytic turnover rates of Rubisco (Yamori & von Caemmerer, 2009). However, the overexpression of both Rubisco and Rca has previously been documented to restore photosynthesis under heat stress, buffering against losses in biomass (Qu *et al*., 2021). Additionally, the temperature optimum of the most abundant isoform of Rca (Rca2β) was increased by 5°C through a single amino acid substitution, thus conferring greater resiliency to Rubisco activation under higher temperatures (Degen *et al*., 2020), offering climate‐proofing avenues for the future. Finally, elevated temperatures can also promote greater photoinhibition (Tan *et al*., 2020), which could negatively affect rates of photosynthetic induction.

Under increased temperatures, the photosynthetic assimilation rate can also be negatively impacted by diffusional limitations, most notably stomata. Stomatal limitations will generally increase with temperature due to an increase in sensitivity to CO_2_ driven by a decline in Rubisco activity and/or higher vapour pressure deficit (VPD) commonly experienced under high air temperatures and stimulating stomatal closure. In these scenarios, stomatal limitations may overtake biochemical limitations of CO_2_ assimilation (Sage & Sharkey, 1987; Lin *et al*., 2021).

When designing an experiment focussed on photosynthetic induction to light under a specific temperature, an initial recommendation would be to mirror the leaf temperature in which the plants are grown. Leaf temperature can be assessed using a thermocouple within a gas analyzer, an infra‐red gun, or using a thermal camera.

It is important to identify key measurement times for determining the impact of a heatwave (or treatment) on a rate of photosynthetic induction. Heatwaves can be acute (short term) or chronic (long term) (Smith & Dukes, 2017). Under chronic heatwaves, induction measurements can also be used to assess the degree of acclimation to heat. Ideally, sequential assessments can be undertaken throughout a heatwave to capture the dynamic response of photosynthesis to increased heat and subsequent acclimation or recovery.

When varying temperature during gas exchange measurements, including photosynthetic induction, it is advisable that the plant and the measuring equipment are briefly acclimated to the change in temperature (20–30 min). To determine the photosynthetic thresholds for optimum or excess temperature, the user can utilize temperature–response curves (Bernacchi *et al*., 2001). For an additional resource on best practices in basic gas exchange measurements, please refer to Busch *et al*. (2024).

Recent methodologies utilizing chlorophyll fluorescence can also support the investigation of photosystem II (PSII) efficiency under a range of temperatures (Ferguson *et al*., 2020). While the temperature changes are more rapid than those applied using a gas exchange cuvette, these assays can provide key information on the critical temperature tolerance of PSII for many leaves at once. Combined with stepwise increases in light, these techniques could facilitate a greater understanding of the relationship photosynthesis has with combined heat and light experienced in the field.

To date, most of the studies that have examined the effects of temperature on Rubisco activation and photosynthetic induction have focussed on transient or short‐term increases in temperature. Considerably less is known about photosynthetic induction – or photosynthesis under fluctuating light – within the context of heat‐acclimated plants. These conditions present an important opportunity to understand how limitations to photosynthetic induction are impacted and what this means for plant productivity under hotter conditions.

Photosynthetic induction is also impacted by low temperatures or cold stress. As early as 1937, slower rates of photosynthetic induction were observed for *Hordeum vulgare* (barley) at low temperatures (McAlister, 1937). Similar results have been shown recently in other species ranging from tomato (*Solanum lycopersicum*) to shade‐tolerant and shade‐intolerant trees in lowland tropical rainforests (Kaiser *et al*., 2017; Kang *et al*., 2020). Both chilling (0°C < T < 15°C) and freezing (*T* < 0°C) stresses have severe implications for limiting CO_2_ assimilation and growth. For C_4_ species, decreasing leaf temperature from 15°C to 5°C increased the duration of photosynthetic induction from 40 min to 2–5 h in *Spartina anglica* (Long, 1976). While in *Z*ea *mays*, photosynthetic induction at 5°C took five times as long as the same induction at 15°C (Long, 1983a,b). Generally, the lower the temperature, the slower the response of photosynthetic induction.

Under colder temperatures, the fluidity of the plasma membrane declines, inhibiting the mobilization of hydrophobic proteins, redox homeostasis, and the repair of the D1protein – a key subunit of PSII. This ultimately restricts the electron transport rate (Aro *et al*., 1990; Allen & Ort, 2001; Mishra *et al*., 2019). Additionally, the mobilization of CBB cycle enzymes – including Rubisco – also declines, leading to an imbalance between light energy absorbed and utilized, leading to photoinhibition (Horton, 2012; Khanal *et al*., 2017). Low temperatures also promote stomatal closure and inhibit stomatal opening through the production of abscisic acid, restricting CO_2_ uptake and therefore limiting photosynthetic induction (Charrier, 2021; Guo *et al*., 2021). At freezing temperatures, plants are also likely to experience dehydration stress caused by extracellular and mesophyll ice formation (Hacker *et al*., 2008), while the presence of ice can physically block the photosynthetic reaction centres (Pospíšil *et al*., 1998). To our knowledge, there are no studies that have looked at the biochemical limitations to carbon fixation in response to an increase in light under low temperature. Furthermore, as with heat, further effort needs to be placed in characterizing photosynthetic induction inter‐ and intraspecies variation for plants acclimated to lower temperatures.

Finally, whatever leaf temperature is being investigated for changes in the speed of photosynthetic induction, the user should consider the temperature‐specific functions predicting Rubisco kinetic properties at that temperature (Bernacchi *et al*., 2001). A worked example of this temperature correction can be found in Dataset S1.

### 3. Water availability and humidity

Less emphasis has been placed on understanding the role of water availability and humidity on photosynthetic induction response (Lawson & Morison, 2004; Lawson & Blatt, 2014; Kaiser *et al*., 2015). Both water availability and humidity play an essential role through mediating the rate of stomatal responses, which can in turn affect the biochemical and diffusional limitations during photosynthetic induction. As the air surrounding the leaf becomes drier, transpiration rates increase, stimulating stomatal closure (and therefore limiting CO_2_ uptake) to prevent excessive water loss (Sakoda *et al*., 2022). However, low water availability or drought can affect photosynthesis beyond limitations caused by stomatal closure. For example, Flexas & Medrano (2002) demonstrated that drought stress affects photosynthetic processes by decoupling adenosine triphosphate (ATP) synthesis, which in turn affects RuBP regeneration, leading to a decreased amount of RuBP. Consequently, this decrease in available RuBP can lead to a decrease in the rate of photosynthesis and the subsequent rate of response of photosynthetic induction. As a result, the water status of the plant needs to be taken into consideration when designing experiments related to photosynthetic induction, as any water stress may reduce the rate of photosynthetic induction.

While relative humidity (RH) describes, as a percentage, how saturated the air is with moisture, VPD quantifies the difference between the measured moisture in the air and what the moisture would be if the air were saturated at a given pressure at a set leaf temperature. In other words, 50% RH at 25°C and 50% RH at 30°C are not the same VPD. Vapour pressure deficit enables a more accurate assessment of the evaporative demand of the atmosphere surrounding the leaf.

As expected, a lower air humidity creates a larger VPD, resulting in lower stomatal conductance to water (*g*_sw_) as the leaf closes its stomates to reduce potential water loss (Pantin & Blatt, 2018). Stomatal closure limits CO_2_ diffusion into the intracellular spaces, resulting in lowered intercellular CO_2_ concentration (*C*_i_). This closure may not be uniform, leading to stomatal patchiness, when areas of stomata remain open on the leaf while in other areas, stomata remain fully closed (Terashima *et al*., 1988; Mott & Buckley, 2000), decreasing the effective measurement area for photosynthesis and leading to inaccurate estimations of *C*_i_. Recent work has also shown that under mild‐to‐high VPD, water content within the substomatal cavity has been overestimated, leading to errors in the calculation of stomatal conductance to CO_2_ diffusion (*g*_sc_), *C*_i_, and mesophyll conductance (*g*_m_; Cernusak *et al*., 2018; Wong *et al*., 2022; Marquez *et al*., 2025). Márquez *et al*. (2023) propose a method to estimate the contribution of patchiness and unsaturation of the substomatal cavity; however, these measurements require a gas exchange system, which can independently measure ad‐ and abaxial surfaces of the leaf.

Previously, it has been shown that low humidity can reduce the rate of photosynthetic induction (Kaiser *et al*., 2017). Furthermore, with low *C*_i_, a decline in Rubisco activation state could result in slower Rubisco activation during lightflecks (Kaiser *et al*., 2015). Humidity within a crop canopy can vary due to different microclimates caused by changes in wind, light availability, and soil moisture. Taking into consideration the changes in VPD or RH throughout a plant canopy would also be relevant to understanding how photosynthetic induction is affected by leaf positioning within the canopy. As such, more work could be focussed on replicating and investigating the multifaceted environment experienced by the leaf undergoing photosynthetic induction.

A common pitfall in measurements of photosynthetic induction, or measurements of photosynthesis under fluctuating light in general, is issues with controlling humidity within the measuring cuvette. One way to navigate this, especially in newer versions of IRGAs (such as the LI‐6800), is controlling for VPD instead of RH within the cuvette. However, if you are using an older system in which controlling humidity is not as precise (such as with an LI‐6400 or a custom‐built chamber), then it is advisable to use equipment such as a dewpoint generator to directly control air water content or to avoid changing the humidity throughout the measurement. Without a dewpoint generator or using a gas exchange system with limited VPD control, a carboy system can be employed to buffer large changes in ambient humidity during measurements, but this set‐up is not ideal for comparing inductions at different VPDs.

A step increase in light intensity can lead to a temporary shift in RH as temperatures rise, stomata open, and transpiration increases. Furthermore, condensation within the measurement cuvette can be a risk when measuring at a chamber temperature that is higher than ambient temperature, as air passing through the leaf chamber is then cooled once exposed to ambient temperature in the sample cell.

The optimal RH is dependent upon the measured species and its growing conditions; however, this is often between 50 and 70% (0.8–1.5 kPa). Finally, data points that increase or decrease sharply around the change in irradiance are often an artefact of the measurement, rather than physiologically representative of plant performance.

### 4. CO_2_ concentration

Transient increases in [CO_2_], in which [CO_2_] is elevated for the duration of the induction measurement, significantly increase the rate and amount of CO_2_ assimilated during photosynthetic induction as well as the eventual steady‐state rates of photosynthesis in plants such as Arabidopsis, tomato, soya bean, rice, and wheat (Kaiser *et al*., 2016, 2017; Soleh *et al*., 2016; Yamori *et al*., 2020; Acevedo‐Siaca *et al*., 2020, 2021a,b; Kang *et al*., 2021). Conversely, and expectedly, transient decreases in [CO_2_] reduce the total amount of CO_2_ assimilated during induction and final steady‐state rates of *A* (Kaiser *et al*., 2016; 2017; Soleh *et al*., 2016; Acevedo‐Siaca *et al*., 2020). Interestingly, transient increases in [CO_2_] do not always significantly increase or change the speed of photosynthetic induction (Acevedo‐Siaca *et al*., 2020; Kang *et al*., 2021). That is to say, the speed of induction as quantified by determining 50 and 90% of final steady‐state *A* (Fig. 1) sometimes does not significantly differ between measurements made at ambient [CO_2_] and those made at elevated [CO_2_] (Kang *et al*., 2021). However, in some *japonica* rice and poplar accessions, the speed of photosynthetic induction increased significantly under transient elevated [CO_2_] (Tomimatsu *et al*., 2019). Similar results have been shown in other studies focussed on tree species (Tomimatsu & Tang, 2016). As a whole, these results suggest that limitation by stomata and biochemistry is impacted by CO_2_ concentration and can differ between species and individual accessions.

When measuring photosynthetic induction at different, short‐term levels of [CO_2_], the leaf should be allowed to reach steady state at the [CO_2_] used for the measurement before starting the induction curve. This can be performed at the same time that the leaf is being exposed to low‐light conditions, which may take anywhere between 30 and 60 min. Not exposing the leaf long enough to the transient change in [CO_2_] used for the measurement can introduce noise in the data and affect the rates of CO_2_ assimilation during photosynthetic induction and any limitations that may be estimated later from those data. For example, a sudden change in [CO_2_] can also cause oscillations in *A*, which can be related to triose phosphate use (TPU) limitation or PSI acceptor‐side limitations (McClain & Sharkey, 2023). The leaf should be exposed long enough to ensure that stomata have sufficiently opened or closed in response to the change in [CO_2_] and *A* allowed to stabilize.

In addition to transient, or short‐term exposure to changes in [CO_2_], plants also respond and acclimate to long‐term changes in exposure to [CO_2_]. To achieve acclimation, plants are grown at a [CO_2_] that differs from current atmospheric [CO_2_], either higher or lower. Most attention has been focussed on measuring photosynthetic induction at either ambient [CO_2_] or during transient changes in [CO_2_]; therefore, much more needs to be known about photosynthesis under dynamic light conditions at acclimated elevated [CO_2_].

The rate of photosynthetic induction and final steady‐state is impacted by acclimation to elevated CO_2_, although the response may be species‐ and genotype‐dependent. For example, poplar genotypes grown under elevated [CO_2_] conditions saw a significant increase in the speed of photosynthetic induction relative to ambient [CO_2_] as quantified by the time constant of *A* to reach 50 and 90% of full induction (Tomimatsu & Tang, 2012). These results coincided with higher initial IS in the leaves of acclimated plants (Tomimatsu & Tang, 2012). Additionally, the faster induction response was independent of the different stomatal behaviour in the poplar genotypes, including differences in stomatal opening and initial stomatal conductance rates before exposure to high light (Tomimatsu & Tang, 2012). Meanwhile, the speed of induction was not significantly impacted in rice and wheat plants acclimated to high [CO_2_] (Kang *et al*., 2021). Furthermore, rice and wheat plants that were acclimated to elevated [CO_2_] had higher *C*_i_ but similar induction speeds as plants grown at ambient [CO_2_], suggesting that they were more heavily limited by biochemistry than stomata during their induction (Kang *et al*., 2021).

Differences in induction rates between plants acclimated to elevated atmospheric [CO_2_] and ambient atmospheric [CO_2_] could be due to differences in Rubisco and Rca content in leaves (Yamori *et al*., 2012; Carmo‐Silva *et al*., 2013). Rubisco content is known to decrease in response to carbohydrate accumulation in leaves under conditions of long‐term exposure to CO_2_ (Long *et al*., 2004). Additionally, increased *C*_i_ due to elevated [CO_2_] increases the rate of ATP consumption, leading to a decrease in ATP:ADP (Gardeström & Wigge, 1988). Previously, the ratio of ATP:ADP was identified as a likely determinant of Rca activity (Crafts‐Brandner & Salvucci, 2000). Consequently, Rubisco activation at elevated [CO_2_] decreases in response to decreased activation by Rca due to lower ATP : ADP ratios as opposed to Rubisco deactivation as seen at higher temperatures (Crafts‐Brandner & Salvucci, 2000). This is despite Rca being upregulated in some plants under elevated [CO_2_], which may also further reduce Rubisco content (Fukayama *et al*., 2012, 2018). Previously, Kang *et al*. (2021) demonstrated that rice and wheat genotypes acclimated to elevated [CO_2_] had lower steady‐state *A* rates before and after photosynthetic induction relative to plants grown in ambient [CO_2_] that were measured under transient elevated [CO_2_]. This discrepancy in photosynthetic capacity could be due to differences in Rubisco and its activation, although characterization of Rubisco and Rca activity and activation state would need to be examined to confirm this.

Overall, previous work suggests that the response of photosynthetic induction to both short‐term and long‐term exposure to elevated [CO_2_] may be species‐ and, in some cases, genotype‐dependent (Tomimatsu & Tang, 2016). Furthermore, limitations to induction may also change depending on [CO_2_] regime. In genotypes where the speed of induction significantly increased with elevated [CO_2_], it is possible that previous diffusional limitations were reduced with enrichment of CO_2_. Meanwhile, those that do not respond to CO_2_ enrichment may be more limited by long‐term biochemical limitations within the leaf (Kaiser *et al*., 2015). These potential differences must be taken into consideration in future measurements to avoid generalizations of entire species. As the current literature suggests that photosynthetic induction responses are not uniform, future studies could incorporate as many diverse genotypes as logistically possible to better understand patterns in induction responses. Finally, most studies focussed on photosynthetic induction under elevated [CO_2_], in both short and long terms, have concentrated on tree species, while additional emphasis could be placed on crop species in the future as a means to assess the links between yield improvement and speed of photosynthetic induction (Tomimatsu & Tang, 2016).

## III. Quantifying photosynthetic induction: calculating limitations and rate of response

Photosynthesis in fluctuating light environments can be categorized into three different processes: photosynthetic induction, postillumination CO_2_ fixation, and postillumination CO_2_ burst until achieving steady‐state rate (Kaiser *et al*., 2015). The postillumination CO_2_ fixation and burst refer to response after a stepwise decrease in light intensity following photosynthetic induction and will not be covered here. Within the process of photosynthetic induction, there exists the opportunity to understand the process in greater detail by characterizing: the diffusional limitation by stomata and mesophyll; the biochemical limitation; the speed of induction; and maximum rates of carboxylation (*V*_cmax_) and electron transport (*J*_max_) during induction in real time through the construction of ‘dynamic’ *A*/*C*_i_ curves. The ‘Diffusional limitations’ section aims to compile existing models that have been developed and deployed in the analysis of the induction curves, to better understand the underlying processes that characterize the low‐ to high‐light response. Many of these models have been modified in different studies to suit the objectives of the research. However, here we aim to show the different options available in the analyses of photosynthetic induction data and when they may be relevant to use based on collected data – with the aim of bringing a greater consistency to future studies. The presented models were originally developed to characterize photosynthetic induction in C_3_ plants. Some models for C_4_ photosynthetic induction are described in Wang *et al*. (2021), although this is a field of work that is still developing and requires further attention.

A worked example of the methodologies discussed in this section is available as part of Dataset S1.

### 1. Diffusional limitations

Following Urban *et al*. (2007), adapted from Woodrow & Mott (1989), this model removes stomatal limitations by assuming a constant *C*_i_ and estimates a corrected CO_2_ assimilation from plants measured from completely dark‐adapted plants:

(Eqn 1) $$A^{*}=\frac{\left(A+R_{D}\right)\left(C_{\text{if}}-\Gamma^{*}\right)}{\left(C_{i}-\Gamma^{*}\right)}-R_{D}$$

where A* represents the transient, *C*_i_‐corrected CO_2_ assimilation during photosynthetic induction, *A* is the actual CO_2_ assimilation rate at a point in time during induction, *R*_D_ is the steady‐state rate of dark respiration, *C*_if_ is the final intercellular CO_2_ concentration at the end of induction, *C*_i_ is the intercellular CO_2_ concentration at a point in time, and Γ* is the CO_2_ compensation point in the absence of photorespiration. To acquire the values for some of these parameters, such as *R*_D_ and Γ*, CO_2_–response curves (*A*/*C*_i_ curves) need to be measured and fitted. For a review on measuring and fitting *A*/*C*_i_ curves, please refer to Busch *et al*. (2024). Otherwise, species constants can be utilized. Limitation by stomata (LS) can then be estimated (Urban *et al*., 2007):

(Eqn 2) $$\mathrm{LS}=\frac{A^{*}-A}{A_{f}+R_{D}}$$

where *A* is CO_2_ assimilation at a point in time and *A*_f_ is the final and maximum CO_2_ assimilation rate at the end of the induction period during high irradiance.

Another method to calculate limitation by stomata or diffusional limitation is by implementing equations adapted by Kaiser *et al*. (2017). CO_2_ assimilation can be corrected for both stomatal and mesophyll limitations as follows:

(Eqn 3) $$A_{C_{a}}^{*}=A\times \frac{\min\left\{A_{c}\left(C_{a}\right),A_{j}\left(C_{a}\right),A_{p}\left(C_{a}\right)\}\right.}{\min\left\{A_{c}\left(C_{c}\right),A_{j}\left(C_{c}\right),A_{p}\left(C_{c}\right)\}\right.}$$

where *A*_c_, *A*_j_, and *A*_p_ are all estimated following the Farquhar–von Caemmerer–Berry model (Farquhar *et al*., 1980), where *R*_D_ is CO_2_ evolution by mitochondria in the light.

(Eqn 4) $$A_{c}\left(C_{a}\right)=V_{c,\max}\;\left(\frac{C_{a}-\Gamma^{*}}{C_{a}+K_{c}\times \left(1+\frac{o}{K_{o}}\right)}\right)-R_{D}$$

(Eqn 5) $$A_{j}\left(C_{a}\right)=J\;\left(\frac{C_{a}-\Gamma^{*}}{4\times C_{a}+8\times \Gamma^{*}\;}\right)-R_{D}$$

(Eqn 6) $$A_{p}\left(C_{a}\right)=3\times \mathrm{TPU}-R_{D}$$

Chloroplastic CO_2_ partial pressure (*C*_c_) can be estimated using a value for mesophyll conductance (*g*_m_):

(Eqn 7) $$C_{c}=C_{i}-\frac{A}{g_{m}}$$

There are two common methods for estimating *g*_m_: the variable *J* method (Harley *et al*., 1992); and the isotope discrimination method (Evans *et al*., 1986). The former utilizes combined gas exchange and chlorophyll fluorescence measurements, while the latter utilizes carbon isotope discrimination methods. For a recent review into the methods to estimate *g*_m_ and the response of *g*_m_ to environmental changes, see Márquez & Busch (2024).

The percent limitation by stomata (LS) can then be estimated (Kaiser *et al*., 2017):

(Eqn 8) $$\mathrm{LS}=\frac{A_{C_{a}}^{*}-A}{A_{f}-A_{i}}\times 100$$

In cases where it is not possible to acquire values for *C*_c_, *C*_i_ can be substituted for *C*_c_ in Eqn 3. However, this will only remove stomatal limitation and will not account for limitation by diffusion across the mesophyll during induction; therefore, it would be quantifying overall limitation by diffusion (LD).

If it is not possible to correct CO_2_ assimilation for stomatal conductance (*g*_s_), an alternative is to estimate the percent limitation by stomata. However, this assumes that induction is limited by *g*_s_ until reaching at least 95% of total *A*, which may not be physiologically true in all or most cases (McAusland *et al*., 2016).

(Eqn 9) $$\text{Limitation}\;\left(\%\right)=\frac{\int_{0}^{t}\left(A_{\max}-A\right)}{\int_{0}^{t}\left(A_{\mathrm{tot}}\right)}$$

where $\int_{0}^{t}\left(A_{\max}-A\right)$is the integral of the difference between the maximum potential *A* (*A*_max_) and the observed *A* from the beginning of the induction curve until time *t* where *A* reached 95% of steady‐state *A*. $\int_{0}^{t}\left(A_{\mathrm{tot}}\right)$ is the maximum integral for *A* over a total period of time (e.g. 30 min, 40 min, and 1 h), where tot represents the total amount of time of the measurement. Calculating the ratio utilizing $\int_{0}^{t}\left(A_{\mathrm{tot}}\right)$ allows for a normalization of *g*_s_ limitation over the duration of the measurement.

### 2. Rubisco properties and biochemical limitations

In addition to estimating diffusional limitations during photosynthetic induction, estimations can also be made about properties related to Rubisco, such as the time constant of the activation of Rubisco, the concentration of Rca, and removing stomatal limitation to examine biochemical limitation.

#### Time constant of Rubisco (τ_Rubisco_ )

The time constant of Rubisco activation (τ_Rubisco_) catalysed by Rca can be estimated following the model developed by Woodrow & Mott (1989). τ_Rubisco_ refers to the time it takes for Rubisco to be activated during photosynthetic induction:

(Eqn 10) $$\tau_{\text{Rubisco}}=-\frac{1}{\text{slope}}$$

The time constant of Rubisco activation is estimated from the slope of the linear portion of a semilogarithmic plot of photosynthesis over time. The slope is determined through linear regression of the linear portion of the plot (typically beginning 2–3 min after the start of induction) (Mott &Woodrow, 1993; Woodrow & Mott, 1989; Wang *et al*., 2021). The slope is estimated from the following equation, where A*_f_ is final CO_2_ assimilation corrected for *C*_i_ and A* is CO_2_ assimilation at a point in time corrected for *C*_i_:

(Eqn 11) $$\ln\left(A_{f}^{*}-A^{*}\right)$$

#### Rca concentration – [Rca]

(Eqn 12) $$\left[\mathrm{Rca}\right]=\frac{k}{\tau_{\text{Rubisco}}}$$

Afterwards, the concentration of Rubisco activase [Rca] can be estimated utilizing the value for τ_Rubisco_, where *k* is a constant equal to 216.9 min mg m^−2^ (Mott & Woodrow, 2000; Wang *et al*., 2021).

#### Removing limitation by stomata to examine biochemical limitation

A simplified method to remove the limitation by stomata is by correcting *A* utilizing the final, steady‐state values of *C*_i_ during high irradiance at the end of photosynthetic induction. This then allows a comparison with raw CO_2_ assimilation values for a simplified evaluation of biochemical limitation. It can be estimated as adapted from Soleh *et al*. (2016) and Acevedo‐Siaca *et al*. (2020):

(Eqn 13) $$A^{*}=A\times \frac{C_{\text{if}}}{C_{i}}$$

where *A* is CO_2_ assimilation at a point in time during induction, *C*_i_ is intercellular CO_2_ concentration at a point in time during induction, and *C*_if_ is the final or steady‐state value of *C*_i_ at the end of induction. In previous studies, the value for *C*_if_ has been selected and treated as a constant for all surveyed genotypes or species measured, for example 300 μmol mol^−1^.

### 3. Quantitative limitation analysis

This approach assumes there are three relative limitations that act on total net photosynthesis at any one time: stomatal (*L*_s_), mesophyll conductance (*L*_m_), and biochemical (*L*_b_). While it could be argued this methodology simplifies the complexities associated with limited *A*, it does provide a broad assessment of the relative contributions of these limitations between plants. Proposed by Jones (1987), Grassi & Magnani (2005), Tomás *et al*. (2013), and Lei *et al*. (2022), the sum of these limitations can be described as Eqn 14:

(Eqn 14) $$L_{s}+L_{m}+L_{b}=1$$

These different components can be calculated as follows (Eqns (Eqn 15), (Eqn 16), (Eqn 17)):

(Eqn 15) $$L_{s}=\left(\left(g_{\mathrm{tot}}/g_{s}\right)\times \left(\left(\partial A_{N}\right)/\left(\partial C_{c}\right)\right)\right)/\left(g_{\mathrm{tot}}+\left(\left(\partial A_{N}\right)/\left(\partial C_{c}\right)\right)\right)$$

(Eqn 16) $$L_{m}=\left(\left(g_{\mathrm{tot}}/g_{m}\right)\times \left(\left(\partial A_{N}\right)/\left(\partial C_{c}\right)\right)\right)/\left(g_{\mathrm{tot}}+\left(\left(\partial A_{N}\right)/\left(\partial C_{c}\right)\right)\right)$$

(Eqn 17) $$L_{b}=g_{\mathrm{tot}}/\left(g_{\mathrm{tot}}+\left(\left(\partial A_{N}\right)/\left(\partial C_{c}\right)\right)\right)$$

where *A*_N_ represents net photosynthetic assimilation, *C*_c_ is the CO_2_ concentration in the chloroplast (Eqn 7), *g*_s_ is the stomatal conductance, *g*_m_ is mesophyll conductance, and *g*_tot_ is the total conductance to CO_2_ from ambient air to chloroplasts, as determined by Eqn 18:

(Eqn 18) $$1/g_{\mathrm{tot}}=1/g_{s}+1/g_{m}$$

To estimate $\left(\partial A_{N}\right)/\left(\partial C_{c}\right)$, the slope of the response of *A*_N_‐*C*_c_ over a range of *C*_c_ between 50 and 100 μmol mol^−1^ can be measured (Tomás *et al*., 2013). To estimate *g*_m_, the variable *J* method (Harley *et al*., 1992) can be used or *g*_m_ can be estimated anatomically (Evans, 2021).

Using the method proposed by Sakoda *et al*. (2021), biochemical limitations on *A*_N_ can be assumed to be either RuBP carboxylation (Eqn 19 – *A*_c_) or RuBP regeneration‐limiting conditions (Eqn 20 – *A*_r_) as developed by Farquhar *et al*. (1980):

(Eqn 19) $$A_{c}=\frac{V_{\text{cmax}}\left(C-\Gamma^{*}\right)}{C+K_{c}\left(1+O/K_{o}\right)}-R_{d}$$

(Eqn 20) $$A_{r}=\frac{J\left(C-\Gamma^{*}\right)}{4C+8\Gamma^{*}}-R_{d}$$

where *V*_cmax_ is the maximum rate of RuBP carboxylation, *C* and *O* are the CO_2_ and O_2_ concentrations, and *K*_c_ and *K*_o_ are the Michaelis constants for CO_2_ and O_2_, respectively. *J* is the rate of whole chain linear electron transport. *K*_c_, *K*_o_, and Γ* can be calculated using leaf temperature response functions described by Bernacchi *et al*. (2001).

### 4. Photosynthetic induction state

The photosynthetic IS refers to the proportion or percentage of CO_2_ assimilation at a given point in time relative to the final CO_2_ assimilation reached during steady state at high irradiance. For leaves measured directly from dark adaptation (and therefore, having an estimate of steady‐state *R*_D_), it can be estimated as follows, as adapted from Chazdon & Pearcy (1986) in Urban *et al*. (2008):

(Eqn 21) $${\text{IS}}_{t}=\frac{A\left(t\right)-R_{D}}{A_{\max}-R_{D}}\times 100$$

where IS_t_ is the IS at *t* time, *A*(*t*) is the transient CO_2_ assimilation rate after *t* time after initial illumination of the leaf and the beginning of induction, *R*_D_ is steady‐state dark respiration, and *A*_max_ is the rate of light‐saturated CO_2_ assimilation at steady state.

The photosynthetic IS can also be calculated for leaves that have been acclimated to shade (as opposed to darkness) before measuring photosynthetic induction, also adapted from Chazdon & Pearcy (1986):

(Eqn 22) $${\text{IS}}_{t}=\frac{A\left(t\right)-A_{i}}{A_{f}-A_{i}}\times 100$$

where *A*_i_ is the initial *A* during low irradiance and *A*_f_ is the final steady‐state value for *A* during high irradiance (Fig. 1).

### 5. Forgone CO_2_ assimilation

Forgone CO_2_ assimilation refers to the CO_2_ assimilation ‘lost’ (*C*_Loss_) due to the lower rates through induction compared with steady state. It attempts to capture some of the losses due to a lag in photosynthetic efficiency during induction. It can be estimated as adapted from Acevedo‐Siaca *et al*. (2020):

(Eqn 23) $$C_{\text{Loss}}=\left(A_{f}-{\bar{A}}_{t}\right)\times t$$

where *A*_f_ is the steady state or final rate of CO_2_ assimilation and *Ā*_t_ is the average rate across the measured time period from the start of the induction (*t*).

### 6. Quantifying the speed of induction

The speed of induction‐related traits is an important factor that is useful to compare between genotypes and treatments. The time to reach 50 and 90% of the final induction value for a trait of interest (Fig. 1) has been utilized as a method to quantify the speed of induction in many studies examining photosynthetic induction (McAusland *et al*., 2016; Soleh *et al*., 2016; Acevedo‐Siaca *et al*., 2020; De Souza *et al*., 2020). However, this can be adapted to any time constant of interest (e.g. 20 and 63%). Some variation exists in how this parameter is calculated.

#### The rate of induction for A and g_s_

In Urban *et al*. (2008), the time to 90% *A* (*T*_9o A_) or *g*_s_ (*T*_90 gs_) was estimated using the following equation. Although *T*_90 A_ is shown, the variables can be replaced with the values for *g*_s_ to calculate *T*_90_ *g*_s_.

(Eqn 24) $$T_{90}=A_{\mathrm{ip}}{\left(\frac{0.9\;A_{\max}-R_{D}}{0.1\;A_{\max}}\right)}^{\frac{1}{s}}$$

where *A*_ip_ is *A* at the inflection point during induction in which *A* begins to reach steady state, *s* is the initial slope of photosynthetic induction, *A*_max_ is the maximum CO_2_ assimilation rate during steady‐state conditions, and *R*_D_ is steady‐state dark respiration. This equation also assumes that photosynthetic induction is taking place from a fully dark‐adapted condition.

However, the time to 90% *A* or *g*_s_ during induction can also be calculated without taking dark respiration, inflection point, or slope into consideration as performed in De Souza *et al*. (2020) and Acevedo‐Siaca *et al*. (2020, 2021a,b), in which the time to 50 and 90% induction was calculated by directly fitting photosynthetic induction raw data.

#### Maximum slope of *g*_s_ increase to quantify stomatal opening

Stomatal opening can be estimated by evaluating the slope between maximum and minimum *g*_s_ during a stepwise change in irradiance as documented in Vialet‐Chabrand *et al*. (2013) and later, Vialet‐Chabrand *et al*. (2017):

(Eqn 25) $${\mathrm{Sl}}_{\max}=k\times \frac{G_{\max}-G_{\min}}{e}$$

where *G*_max_ and *G*_min_ represent the maximum and minimum steady‐state *g*_s_, respectively, and *k* is a time constant (McAusland *et al*., 2016; Vialet‐Chabrand *et al*., 2017). The value of *k* can vary between 0.000001 and 0.01 s^−1^ but is dependent on the measurement duration and will likely be species‐ and genotype‐dependent (Vialet‐Chabrand *et al*., 2013). For example, previously, a value of 0.00083 s^−1^ was used for *k* in the modelling of the Sl_max_ values (Vialet‐Chabrand *et al*., 2013), but for McAusland *et al*. (2016), a range of values within the 10–60 min of the light step were chosen.

### 7. Constructing ‘dynamic’ *A*/*C*_i_ curves from induction curves

Photosynthetic induction can be measured at low, ambient, and elevated CO_2_ concentrations facilitating the development of constructed or ‘dynamic’ *A*/*C*_i_ curves as shown in Soleh *et al*. (2016), Salter *et al*. (2019), and Acevedo‐Siaca *et al*. (2020). In theory, since CO_2_ assimilation is measured throughout the entire photosynthetic induction process, an individual *A*/*C*_i_ curve can be constructed at each time point. These ‘dynamic’ *A*/*C*_i_ curves can then be fit to determine biochemical and diffusional limitations *in vivo*. Although the measurements may be time‐consuming, they allow biochemical limitations such as the maximum rate of carboxylation (*V*_cmax_) and the maximum rate of photosynthetic electron transport (*J*_max_) to be estimated in real time during induction at each of the measured time points. This then allows a greater understanding of how these limitations change over the course of photosynthetic induction. For example, in soya bean, the use of ‘dynamic *A*/*C*_i_ curves’ allowed for confirmation that the genotypes that had stronger photosynthetic induction responses also had less limitation by *V*_cmax_ over time (Soleh *et al*., 2016). In wheat, dynamic *A*/*C*_i_ curves allowed for comparison among genotypes for *J*_max_ and *V*_cmax_. These comparisons showed different combinations of the two limitations over time that changed dynamically in single genotype (Salter *et al*., 2019). Finally, in rice, *V*_cmax_ was found to be limiting in the genotypes that were surveyed at the beginning of photosynthetic induction (Acevedo‐Siaca *et al*., 2020).

These measurements can be particularly valuable when combined with information about real‐time diffusional and biochemical limitations during changes from low to high light. In addition to allowing the estimation of biochemical limitations, measuring photosynthetic induction at several [CO_2_] allows for the examination of how photosynthetic induction responds to transient changes in [CO_2_].

**Fig. 4:**
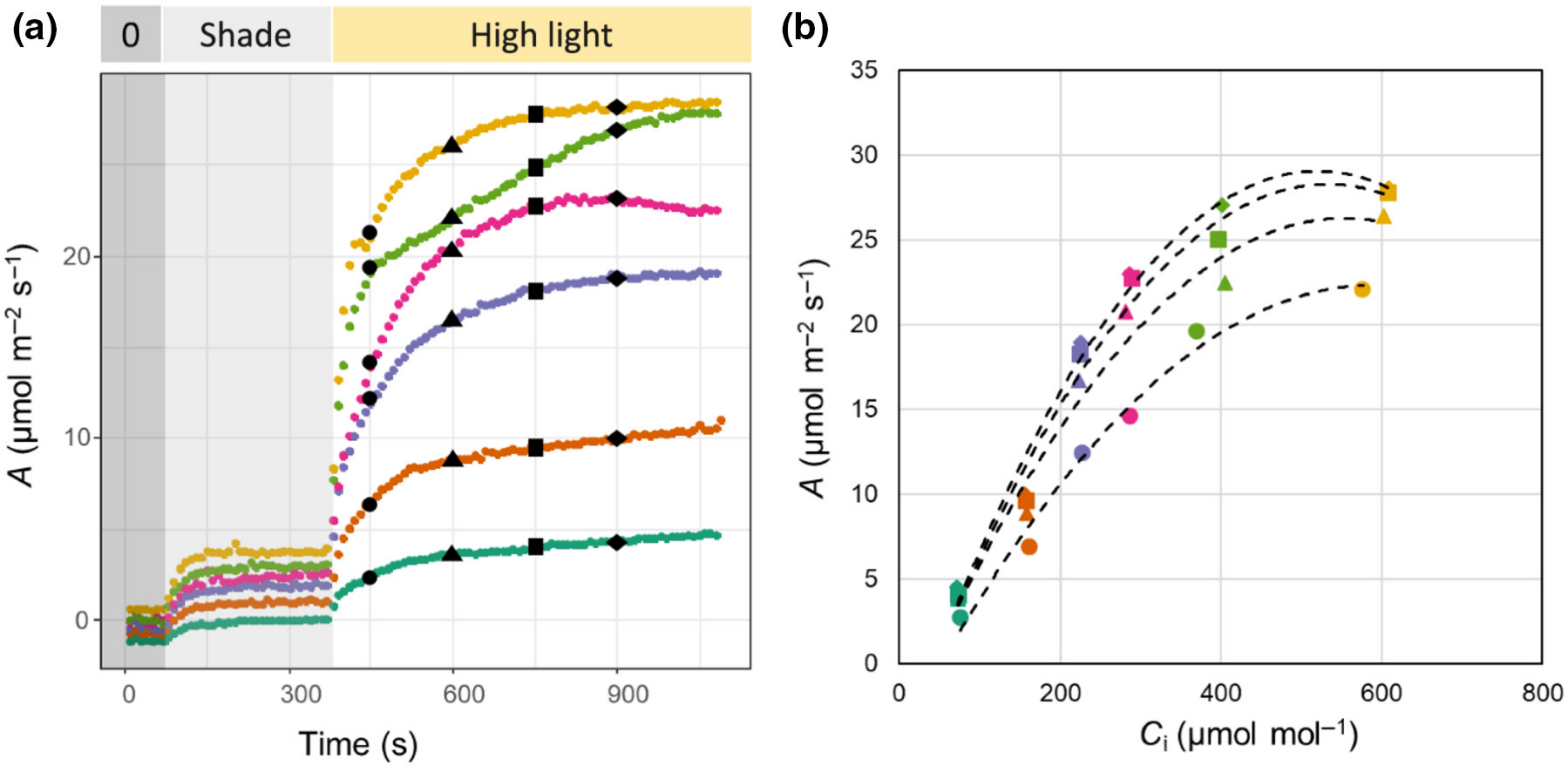
Construction of ‘dynamic’ *A*/*C*_i_ curves from photosynthetic induction curves measured at several [CO_2_] in rice (*Oryza sativa*, cv IR64‐21). (a) An example of the response of CO_2_ assimilation (*A*) to an increase in light intensity from 50 to 1700 μmol m^−2^ s^−1^ photosynthetic photon flux density over time under six different [CO_2_] regimes: 100, 200, 300, 400, 600, and 800 μmol mol^−1^. Black points represent the same point in time (e.g. circles, 450 s; triangles, 600 s; squares, 750 s; diamonds, 900 s) in the induction curves measured at different [CO_2_]. Values for *A* and intercellular CO_2_ concentration (*C*_i_) at the same time point and at different [CO_2_] can then be utilized to construct *A*/*C*_i_ curves (b), which allows examination of how *A*/*C*_i_ curves and related limitations change over time over the course of photosynthetic induction. Here, the same colors represent the same ambient [CO_2_] (teal, 100; orange, 200; purple, 300; magenta, 400; green, 600; and yellow, 800 μmol mol^−1^) where the shape indicates the point in time during the measurement.

‘Dynamic’ *A*/*C*_i_ curves can be constructed in the following way (Fig. 4):

1. Photosynthetic induction is measured at different [CO_2_] in a randomized sequence on the same plant, making sure that a dark adaptation or deep‐shade period takes place between induction curves. The dark adaptation or the deep shade between measurements should ideally last between 30 and 60 min to reduce the likelihood of residual photoprotection, which could influence the next measurement. The leaf should be exposed to the [CO_2_] that will be used during the period of dark or shade adaptation before starting the measurement. This will allow stomata to acclimate to the new [CO_2_] and minimize the risk of oscillations during the measurement due to sudden, stepwise changes in [CO_2_] that could be attributed to TPU limitation or PSI acceptor‐side limitations. Additionally, care should be taken to measure the leaf in the same area for all measurements. To avoid confounding the response of photosynthesis with the order of measurement, the selected [CO_2_] should be randomized per plant. The selected [CO_2_] are representative of the needs of the researcher and the objectives of their study. Consideration should be placed in selecting the CO_2_ concentrations and how many will be measured, as if measurement density is insufficient, it may later be difficult to fit the curves to estimate *V*_cmax_ and/or *J*_max_. For example, too few photosynthetic induction measurements at elevated [CO_2_] may make it difficult to accurately fit *J*_max_. In previous studies, photosynthetic induction was measured at 100, 200, 300, 400, 600, and 800 μmol mol^−1^ to generate ‘dynamic’ *A*/*C*_i_ curves. While these [CO_2_] may allow for a decent fit in most cases for *V*_cmax_, it may be more difficult to estimate *J*_max_ accurately. A response of photosynthetic induction to [CO_2_] may resemble the data depicted in Fig. 4(a) for rice.
2. After photosynthetic induction is measured at different [CO_2_], the construction of the dynamic *A*/*C*_i_ curves can begin. The data for *A* and *C*_i_ are selected at the same time point across all induction curves (Fig. 4a) and then plotted in a conventional *A* vs *Ci* format (Fig. 4b). In theory, an *A*/*C*_i_ curve can be constructed at each of the time points measured during induction (Fig. 4b), provided that the timing of logging of data is the same at all [CO_2_].
3. The *A*/*C*_i_ curves constructed from induction data can then be fit to estimate biochemical parameters across time, for example plotting *V*_cmax_ or *J*_max_ against time. These estimates are useful for comparing performance and limitations between different genotypes and treatments of interest.
4. As a point of comparison, it is good practice to also measure a conventional, steady‐state *A*/*C*_i_ curve per plant or sample (Busch *et al*., 2024).

### 8. Important considerations about calculating limitations

During the photosynthetic induction of dark‐ or shade‐adapted plants, *C*_i_ will be lower at the end of induction (*C*_if_) than at the beginning of induction (Fig. 3) (Soleh *et al*., 2017; Acevedo‐Siaca *et al*., 2020, 2021a,b; Arce Cubas *et al*., 2023). This is applicable to both C_3_ and C_4_ plants, independent of the presence of a carbon‐concentrating mechanism. However, many models that estimate biochemical limitation do not take into account this decrease in *C*_i_ throughout photosynthetic induction relative to initial low irradiance values, making it difficult to fit or estimate biochemical limitation at the beginning of photosynthetic induction. Additionally, most of the pre‐existing models that are utilized to calculate biochemical limitation work on the assumption that CO_2_ assimilation will only increase throughout photosynthetic induction. Yet, these models do not account for situations in which oscillations during photosynthetic induction occur. Under these conditions, the final steady‐state value for *A* may be lower than during the oscillation. Caution should therefore be used when drawing conclusions, as under these situations, it can be difficult to ‘correct’ CO_2_ assimilation to an absence of biochemical limitation.

Oscillations during photosynthetic induction do not always occur and seem to be genotype, leaf water status, and growth condition specific (Yang *et al*., 2005; Acevedo‐Siaca *et al*., 2021a,b; Wen *et al*., 2023). In some cases, oscillations in CO_2_ assimilation during photosynthetic induction are strongly paired to oscillations in stomatal conductance (Acevedo‐Siaca *et al*., 2021a,b; Wen *et al*., 2023). Under these conditions, it is proposed that the oscillations are due to changes in stomatal opening and closing (Wen *et al*., 2023), although the mechanism underlying the stomatal movements during stepwise increases of light is not yet fully understood. However, there are situations in which oscillations in CO_2_ assimilation during photosynthetic induction do not correspond with changes in stomatal opening and closing (Wen *et al*., 2023). In these cases, the changes could be due to the leaf entering TPU limitation quickly and PSI acceptor‐side limitations, which implicate the availability of NADP^+^ and ATP (McClain & Sharkey, 2023; Zhang *et al*., 2024). Furthermore, an initial overshoot can be caused by temporarily excessive available phosphate, in which the leaf performs beyond limitations imposed by TPU and RuBP regeneration, but is unable to exceed limitation by rubisco (McClain & Sharkey, 2023; Zhang *et al*., 2024).

It is not fully possible to characterize limitation to induction by any one equation or by any one number. Many of these equations work under the assumption that the biochemical or diffusional properties that are being corrected for act as constants throughout this dynamic process, which may not be true. Consequently, we think it is important to use these equations or models as guides as to what may be happening during photosynthetic induction, but they may not be fully representative of what is occurring under all conditions. To best understand the limitations to photosynthetic induction by both diffusion and biochemistry, we recommend that photosynthetic induction measurements are always, at minimum, paired with conventional *A*/*C*_i_ curves, which allow for the determination of path‐dependent limitations (Farquhar & Sharkey, 1982; Assmann, 1988). Furthermore, time and equipment permitting, constructed ‘dynamic’ *A*/*C*_i_ curves are the most informative tool for understanding changes in biochemical and diffusional limitations in leaves during induction in real time.

## IV. Future steps

Until now, most photosynthetic induction measurements have been made within the context of singular, stepwise changes in irradiation and on single leaves, rather than on whole plants. Additionally, most measurements have taken place in ambient [CO_2_]. Studies that aim to represent field conditions are essential to translating improvements in photosynthetic induction into improvements in biomass and, potentially, yield for crops. As mentioned previously in the ‘Measurement considerations: designing a photosynthetic induction experiment’ section, we suggest future focus on disentangling the relationship between light and temperature during photosynthetic induction, the role of humidity across the canopy microclimate, and exposure or acclimation to changes in [CO_2_]. Here, we briefly identify additional gaps in our collective understanding on photosynthetic induction, such as the impact of light quality, expanding measurements to plants with varied photosynthetic pathways, and how to increase the scale of non‐steady‐state photosynthetic measurements.

### 1. Light quality

While there have been recent advancements in determining the light environment of a leaf (Burgess *et al*., 2021; Durand & Robson, 2023), further consideration needs to be given to measuring induction under different light spectra. Measurements of CO_2_ assimilation and stomatal conductance are typically made using an IRGA utilizing only red and blue light (typically a mix of 90% red: 10% blue). Measuring photosynthetic induction in response to this specific spectrum ignores the impact of other wavelengths, which could limit processes within the induction response. Indeed, recent studies have shown that the red to blue light ratio may have strong impacts on steady‐state photosynthesis, while minimally influencing photosynthesis under dynamic, non‐steady‐state conditions (Zhang *et al*., 2022). Consequently, further effort needs to be placed into understanding how different light quality affects photosynthetic induction and its limitations, especially in either shaded leaves or canopy understories. This is increasingly possible with off‐the‐shelf IRGAs such as the CIRAS‐4 (PP Systems, Amesbury, MA, USA) or with chamber head additions for the Li‐6800 (6800–03; Li‐COR Environmental, Lincoln, NE, USA), which allow for greater control of the light spectrum within the measuring cuvette.

Red, blue, and green photons are all capable of driving photochemistry once they are absorbed (Smith *et al*., 2017). However, as sunlight penetrates the plant canopy, light intensity attenuates and changes in composition; red (600–700 nm) and blue (400–500 nm) light are predominantly absorbed to drive photochemistry and stimulate guard‐cell opening, respectively (Matthews *et al*., 2020). Leaves in the lower canopies or at ground level are limited by both quantity and quality of light, as light becomes more diffuse and the proportion of green light relative to red light becomes higher (Matthews *et al*., 2020). The presence of green light and the role it may play in how plants acclimate to short‐term dynamic fluctuations and optimize resource use efficiency is currently a subject of active investigation (Aasamaa & Aphalo, 2016; Smith *et al*., 2017; Matthews *et al*., 2020). Previous work suggests that green light may inhibit blue light‐induced stomatal opening under certain situations, such as after applying a pulse of green light immediately after a pulse of blue light or in the morning when potassium is used as an osmoticum in stomatal regulation (Talbott *et al*., 2002, 2006). Additionally, a recent meta‐analysis of green/blue light response suggests that in several horticultural crops, the inclusion of green light in lighting schemes can significantly reduce stomatal conductance without penalizing CO_2_ assimilation, leading to significant increases in intrinsic water use efficiency (Chen *et al*., 2024). Consequently, it is suggested that green light could have water‐saving properties due to reductions in stomatal conductance that could be beneficial in light‐limited conditions, such as within a plant canopy (Smith *et al*., 2017). It is likely that these same effects of light quality that impact steady‐state photosynthesis measurements would also impact photosynthesis under non‐steady‐state conditions, although this is not yet known. Within the context of fluctuating light conditions, stomata that are exposed to a greater proportion of low‐intensity green light may acclimatize to these conditions, reducing their rate of opening in response to lightflecks under certain conditions. Consequently, this may directly limit the rate of photosynthetic induction through limiting CO_2_ diffusion.

### 2. Increasing focus on photosynthetic induction in different photosynthetic pathways

Most of our understanding of fluctuating photosynthesis has come from C_3_ crops and varied studies in trees and shrubs (Table 1). C_2_ photosynthesis, typified by the capture and concentration of CO_2_ released during photorespiration, offers the potential to improve the efficiency of our major C_3_ crops (Lundgren, 2020). While C_2_ plants generally exhibit higher rates of assimilation, less work has focussed on the systematic analysis of the rate of induction to light. Meanwhile, some of the most relevant crops in terms of food security and potential for the generation of biofuels utilize the C_4_ photosynthetic pathway. Under warmer climates and higher light intensities, C_4_ plants are generally considerably more efficient at photosynthesis relative to their C_3_ counterparts due to the separation of the mesophyll and bundle sheath cells and related carbon‐concentrating mechanism.

Several studies suggest that C_4_ plants may be less capable of dealing with fluctuating light conditions and possess less phenotypic plasticity in comparison with C_3_ plants (Sage & McKown, 2006). Reduced biomass production was documented in C_4_ plants grown under fluctuating light conditions in comparison with those grown under steady‐state conditions (Kubásek *et al*., 2013). Additionally, the decrease in biomass was more pronounced in C_4_ plants than under the same treatments in C_3_ plants (Kubásek *et al*., 2013). This decrease is partially believed to be caused by the need to coordinate the C_3_ and C_4_ cycles between mesophyll and bundle sheath cells during processes such as photosynthetic induction (Wang *et al*., 2022). Additionally, some shade‐tolerant C_4_ grasses are unable to maintain high levels of photosynthetic induction or CO_2_ assimilation following irradiance from lightflecks, suggesting that C_4_ plants may be less suited than C_3_ plants to respond to quick changes in light in their environment (Sage & McKown, 2006). In a similar vein, recent work in phylogenetically controlled experiments showed that photosynthetic induction is slower to activate CO_2_ assimilation in C_4_ plants relative to closely related C_3_ and C_3_–C_4_ intermediates (Arce Cubas *et al*., 2023). However, much is still to be known about photosynthetic induction within the context of C_4_ plants, offering ample opportunity for future studies. This is especially promising as variation has been identified for stomatal opening and closing in response to changes in light in species such as maize and sorghum (Pignon *et al*., 2021a,b; Al‐Salman *et al*., 2023; Crawford *et al*., 2024), suggesting that responses may vary between species and genotypes. In addition to expanding our knowledge of photosynthesis in dynamic light environments, understanding the differences in photosynthetic induction between different photosynthetic pathways is crucial to the improvement in the process in diverse plants.

### 3. Increasing the scale of non‐steady‐state photosynthetic measurements

One major limitation of photosynthesis research is the time‐consuming nature of gas exchange measurements. While survey‐style or point measurements may take between 3 and 5 min to complete, measurements that seek to characterize dynamic processes can take upwards of 30 min, not including the necessary periods of shade or dark adaptation. As a result, measuring dynamic photosynthesis is very low throughput, with most studies being limited in the number of genotypes examined. Recently, quick, in‐field chlorophyll fluorescence measurements have been deployed to characterize kinetics related to photosynthetic induction and NPQ on a larger scale in crops (McAusland *et al*., 2019). Chlorophyll fluorescence imaging also allows for higher throughput measurements of plants, while still maintaining controlled conditions for samples. However, effort needs to be placed into understanding how well chlorophyll fluorescence parameters correlate with CO_2_ assimilation values acquired through gas exchange during photosynthetic induction. Additionally, studies have shown that it is possible to estimate complex biochemical limitations to photosynthesis, such as the maximum rate of carboxylation or electron transport, in‐field‐grown plants utilizing remote sensing techniques (Fu *et al*., 2019; Meacham‐Hensold *et al*., 2019). These remote sensing techniques that are neither destructive nor time‐consuming in the initial data collection could eventually be applied to dynamic or fluctuating photosynthesis studies, leading to the eventual development of indices that can predict the speed of induction or limitations to induction. Both approaches can increase the throughput at which photosynthetic induction is evaluated, presenting a way to understand the genetic underpinnings of the process and a potential avenue for inclusion into breeding programs.

## V. Conclusion

Here, we present measurement considerations related to light intensity and exposure, temperature, humidity and plant water status, and CO_2_ concentration when preparing an experiment to measure photosynthetic induction. When designing an experiment focussed on photosynthetic induction, it is crucial that the researcher takes into consideration the light intensity in which the plants have been grown, the duration and intensity of acclimation period to darkness or low light before induction, and the high‐light intensity used during the measurement. In addition to light environment and history, leaves should be allowed to acclimate to changes in temperature and [CO_2_] before the measurement to avoid issues related to stomatal opening or closing. What is decided for each of these factors will ultimately affect the induction curve and what limitations may be found. Consequently, it is crucially important that the protocol used is tailored to the research questions of the experiment. We also compile some of the most used models and equations used to estimate parameters and limitations related to photosynthetic induction. Through this, we hope to provide a ‘toolbox’ that can be used by researchers based on their experimental goals and their availability of equipment.

Finally, we identify current gaps in the literature as it relates to photosynthetic induction. This includes the interaction between light and temperature during induction, the role of microclimate throughout the plant canopy, long‐term acclimation to changes in temperature and [CO_2_], the impact of light spectral quality, measurement in diverse photosynthetic pathways, and barriers to high‐throughput measurements. Through this paper, we hope to provide a reference that can be used when designing and analysing experiments focussed on photosynthetic induction, with the intent of creating more consistency between experiments in the future.

## Competing interests

None declared.

## Author contributions

LAS conducted the experimental work. LM and LAS wrote the manuscript with equal contribution.

## Disclaimer

The New Phytologist Foundation remains neutral with regard to jurisdictional claims in maps and in any institutional affiliations.

## Supporting information


**Dataset S1** An Excel‐based toolkit providing examples of current induction analysis metholdogies.
Please note: Wiley is not responsible for the content or functionality of any Supporting Information supplied by the authors. Any queries (other than missing material) should be directed to the *New Phytologist* Central Office.

## Data Availability

The data that support the findings of this study are available in the Supporting Information of this article (Dataset S1).
